# Glomerular diseases in South African patients on kidney replacement therapy: an analysis of 2022 South African Renal Registry data

**DOI:** 10.1007/s11255-026-05117-0

**Published:** 2026-04-02

**Authors:** Nicolas Allyn Fitchat, Mogamat Razeen Davids, Mogamat-Yazied Chothia

**Affiliations:** Division of Nephrology, Department of Medicine, Faculty of Medicine and Health Sciences, Stellenbosch University, Francie van Zijl Drive, Parow, Cape Town, 7505 South Africa

**Keywords:** Glomerular disease, Kidney failure, South Africa, Diabetes, Lupus nephritis, HIV, Renal registry

## Abstract

**Background:**

Kidney failure is a growing public health burden in sub-Saharan Africa, with limited access to kidney replacement therapy (KRT) and a paucity of epidemiological data, particularly on glomerular disease as an underlying cause. The primary aim of our study was to describe the types of glomerular diseases in prevalent patients receiving KRT in South Africa.

**Methods:**

We conducted a cross-sectional study of South African prevalent patients who were receiving KRT on 31 December 2022 who had glomerular disease as a cause of their kidney failure, as recorded in the South African Renal Registry (SARR). Patients were categorised by glomerular disease subtype, demographic data, KRT modality, sector of care and geographic distribution.

**Results:**

Of the 9342 patients on KRT, 2135 (22.9%) had glomerular disease as a cause of kidney failure. The median age was 55 years (interquartile range: 42–65 years), with a male predominance (56.9%). Secondary glomerular diseases accounted for 67.9% and diabetic nephropathy (56.1%) was the most common, followed by human immunodeficiency virus-associated nephropathy (4.8%) and lupus nephritis (4.5%). Of the primary glomerular diseases, focal segmental glomerulosclerosis (7.7%) was the most common, followed by IgA nephropathy (5.3%), rapidly progressive glomerulonephritis (4.8%) and mesangiocapillary glomerulonephritis (3.8%).

**Conclusions:**

Nearly one-quarter of patients receiving KRT in South Africa have glomerular disease as a cause of their kidney failure, with secondary forms predominating. The high prevalence of diabetic nephropathy underscores the substantial contribution of diabetes mellitus to the burden of kidney failure. Marked disparities were observed between healthcare sectors.

**Supplementary Information:**

The online version contains supplementary material available at https://doi.org/10.1007/s11255-026-05117-0.

## Introduction

Kidney failure is a major public health problem affecting an estimated 844 million people worldwide and of those, 3.9 million are on kidney replacement therapy (KRT) [1]. Due to the lack of renal registries in sub-Saharan Africa (SSA), there are very limited data on the incidence, prevalence and causes of kidney failure in this region [2–4]. The available data indicates that there is limited access to KRT in most developing countries [4].

South Africa operates a two-tier healthcare system consisting of public and private sectors. Only about 15% of the population receives care in the private sector, according to the Council for Medical Schemes, leaving 85% reliant on the resource-limited public sector [5]. Despite serving most of the population, the public sector receives only 20% of national health spending. Consequently, KRT––including haemodialysis, peritoneal dialysis and transplantation––must be rationed in the public sector, while those with medical insurance have near-guaranteed access. The 2022 South African Renal Registry reported 9342 patients on KRT, with an overall treatment rate of 151 per million population (pmp). Treatment rates were markedly higher in the private sector—771 pmp, 17 times the public-sector rate of 45 pmp [6].

Six South African studies describe patterns of glomerular disease. Two Cape Town centres (Tygerberg Hospital and Groote Schuur Hospital) reported similar findings, with mesangiocapillary glomerulonephritis (MCGN) the most common primary glomerulonephritis and lupus nephritis the most frequent secondary form [4, 9].

In contrast, studies from Gauteng and the Free State show focal segmental glomerulosclerosis (FSGS) predominance. Universitas Hospital (Bloemfontein) found glomerular disease in 19.9% of 1216 biopsies, with FSGS most common [10]. A 30 year review from Chris Hani Baragwanath similarly identified FSGS as the leading primary disease and lupus nephritis as the predominant secondary condition, followed by human immunodeficiency virus (HIV)-associated nephropathy (HIVAN) [11]. Reviews from Helen Joseph and Charlotte Maxeke hospitals also showed secondary disease dominance; lupus nephritis and HIV-associated glomerulopathies were most frequent, with FSGS the leading primary disorder [12, 13].

Regional variation likely reflects population differences, particularly ethnicity [14]. MCGN appears more common in people of mixed ancestry in Cape Town [15], and widespread methamphetamine use (“tik”)—linked to MCGN—may contribute to its higher prevalence there [16].

Among people living with HIV, the spectrum is evolving. With the introduction of antiretroviral therapy (ART), glomerular-dominant lesions have decreased and tubulointerstitial disease has become more prominent [17].

This study aimed to describe the burden and spectrum of glomerular diseases as a cause of kidney failure in South African patients receiving KRT during 2022.

## Methods

We performed a cross-sectional analysis of SARR data for all patients receiving KRT on 31 December 2022 whose kidney failure was attributed to glomerular disease. The primary outcome was to describe the frequency and types of glomerular diseases. Secondary outcomes included comparisons by provinces, healthcare sector (public vs. private), ethnicity and sex.

The SARR is a national database of patients receiving KRT (dialysis or transplantation) in South Africa. It was re-established in 2010 and first reported data from 2012 in its 2014 annual report, replacing an earlier registry that ceased in 1994, and has been updated annually since then. The registry includes all patients on chronic dialysis or with a kidney transplant in both the public and private sectors. SARR is owned and overseen by the South African Nephrology Society, incorporated as a non-profit entity, with governance provided by a dedicated Registry Committee and directors drawn from the nephrology community. Data are collected and updated annually by designated data capturers from treatment centres nationwide, with ethical oversight provided through Stellenbosch University’s Health Research Ethics Committee.

We obtained de-identified South African Renal Registry data for all patients receiving KRT who had glomerular disease documented as their cause of kidney failure as of 31 December 2024. Variables included age, sex, ethnicity, diabetes status, HIV status, province, health sector, glomerular disease diagnosis and biopsy status. The dataset was supplied in Excel format.

Inclusion criteria were all patients with a primary kidney diagnosis of glomerular disease, either based on kidney biopsies or clinical diagnosis as reported by the treating KRT centre. An anonymised dataset containing the pertinent data relating to this study was provided by the SARR.

The SARR uses the diagnostic codes of the European Renal Association (ERA) Registry. [18] From these diagnoses, the different glomerular diseases were grouped into two categories, namely primary and secondary.

Primary glomerular diseases included the following diagnoses: GN-no histology, FSGS, GN-histologically indeterminate, adult nephrotic syndrome-no histology, IgA nephropathy (IgAN), rapidly progressive GN (RPGN), MCGN, congenital nephrotic syndrome, mesangial proliferative GN, membranous nephropathy (MN)–idiopathic, Alport’s syndrome, congenital nephrotic syndrome-FSGS, congenital nephrotic syndrome-Finnish type, minimal change disease (MCD), nephrotic syndrome of childhood, renal amyloid, IgM-associated nephropathy and immunotactoid / fibrillary nephropathy.

Secondary glomerular diseases included the following diagnoses: diabetic nephropathy (DN), HIVAN, LN, GN-secondary to other systemic disease, antineutrophilic cytoplasmic antibody (ANCA)-associated GN, diffuse endocapillary GN, polyarteritis nodosum (PAN), anti-glomerular basement membrane (GBM) disease, FSGS secondary to HIV, AA amyloid, MN-infection associated, FSGS secondary to obesity and MN-drug induced.

The SARR granted permission to analyse their data on prevalent patients for the year 2022, and to report on the patients with glomerular disease as the cause of their kidney failure. An anonymised, password-protected dataset was provided by the SARR. Ethical approval was granted by the Health Research Ethics Committee (HREC) of Stellenbosch University (HREC reference number: S24/05/113).

### Statistical analysis

Analyses were performed using International Business Machines Corporation Statistical Package for the Social Sciences Statistics (IBM SPSS Statistics) version 23.1 (IBM Corp, Armonk, NY, USA). Descriptive data were summarised as counts and percentages. Normality was assessed using the Shapiro–Wilk test. Normally distributed continuous variables were presented as means with standard deviations, whereas non-normal variables were reported as medians with interquartile ranges.

## Results

SARR data showed that 9342 patients were receiving KRT, of whom 2135 (22.9%) had glomerular disease as the underlying cause of kidney failure. Of these, 685 (32.1%) had primary glomerular disease and 1450 (67.9%) had secondary forms. Their demographic and clinical characteristics are summarised in Table 1. The median age was 55 years, with a range of 3–95 years. There was a male predominance (56.9%). The HIV seropositive rate was 10.9% and 60.5% had diabetes mellitus. Nearly three quarters of the patients were receiving treatment in the private sector, and two provinces (the Western Cape and Gauteng) accounted for more than half of the patients.

**Table 1:** Demographic and clinical characteristics of the study cohort, by category of glomerular disease

	Glomerular disease, n (%)	Glomerular disease excluding DN, n (%)	Primary glomerular disease, n (%)	Secondary glomerular disease, n (%)	Secondary glomerular disease excluding DN, n (%)
Totals	2135 (22.9)*	937 (10.0)	685 (7.3)	1 450 (15.5)	252 (2.7)
Male sex	1215 (56.9)	484 (51.7)	388 (56.6)	827 (57)	96 (38.1)
Age (years), median (IQR)	55 (42–65)	44 (34–54)	43 (32–53.5)	59 (50–67)	45 (37–54)
Diabetes mellitus	1292 (60.5)	94 (10)	73 (10.7)	1 219 (84.1)	21 (8.3)
HIV positive	232 (10.9)	148 (15.8)	35 (5.1)	197 (13.6)	113 (44.8)
Ethnicity					
Black	1047 (49.0)	423 (45.1)	255 (37.2)	792 (54.6)	168 (66.7)
Mixed	553 (25.9)	303 (32.3)	258 (37.7)	295 (20.3)	45 (17.9)
Indian/Asian	208 (9.7)	45 (4.8)	34 (5)	174 (12.0)	11 (4.4)
White	299 (14)	147 (15.7)	120 (17.5)	179 (12.3)	27 (10.7)
Other	28 (1.3)	19 (2)	18 (2.6)	10 (0.7)	1 (0.4)
Province					
Western Cape	782 (36.6)	491 (52.4)	430 (62.8)	352 (24.3)	61 (24.2)
Gauteng	478 (22.4)	171 (18.2)	111 (16.2)	367 (25.3)	60 (23.8)
KwaZulu-Natal	286 (13.4)	56 (6)	34 (5)	252 (17.4)	22 (8.7)
Eastern Cape	243 (11.4)	62 (6.6)	36 (5.3)	207(14.3)	26 (10.3)
Free State	140 (6.6)	96 (10.2)	44 (6.4)	96 (6.3)	52 (20.6)
Limpopo	77 (3.6)	15 (1.6)	9 (1.3)	68 (4.7)	6 (2.4)
Mpumalanga	53 (2.5)	23 (2.5)	7 (1)	46 (3.2)	16 (6.3)
North West	53 (2.5)	14 (1.5)	8 (1.2)	45 (3.1)	6 (2.4)
Northern Cape	23 (1.1)	9 (1)	6 (0.9)	17 (1.2)	3 (1.2)
Histologically proven	310 (14.5)	310 (33.1)	168 (24.5)	142 (9.8)	142(56.3)
Healthcare sector					
Private	1539 (72.1)	417 (44.5)	274 (40)	1265 (87.2)	143 (56.7)
Public	596 (27.9)	520 (55.5)	411 (60)	185 (12.8)	109 (43.3)
KRT modality					
HD	1527 (71.5)	465 (49.6)	291 (42.5)	1236 (85.2)	174 (69)
PD	223 (10.4)	153 (16.3)	112 (16.4)	111 (7.7)	41 (16.3)
TX	385 (18)	319 (34)	282 (41.2)	103 (7.1)	37 (14.7)

*SARR* South African Renal Registry, n number, *IQR* interquartile range, *DN* diabetic nephropathy, *HIV* human immunodeficiency virus, *KRT* kidney replacement therapy,* HD* haemodialysis, *PD* peritoneal dialysis *TX* transplant

*Proportion relative to the total SARR population as on 31 December 2024 (n = 9342)

In our cohort with glomerular disease, secondary glomerular disease accounted for the majority (67.9%). Overall, diabetic nephropathy was the most common cause of glomerular disease (56.1%). When we excluded patients with diabetic nephropathy, only 10% of the remaining patients had diabetes mellitus.

Patients with primary glomerular disease (Table 2) had a younger median age than those with secondary glomerular diseases (43 years vs. 59 years); however, the median ages were similar if patients with diabetic nephropathy were excluded (Tables 1, 3).

**Table 2:** Frequencies and proportions of specific primary glomerular diseases

Primary glomerular disease	Frequency	Overall % (n = 2 135)	% of primary GN (n = 685)
Primary	685	32.1	100
Glomerulonephritis—no histology	378	17.7	55.2
FSGS	53	2.5	7.7
Glomerulonephritis—histologically indeterminate	49	2.3	7.2
Adult nephrotic syndrome—no histology	42	2.0	6.1
IgAN	36	1.7	5.3
RPGN	33	1.5	4.8
MCGN	26	1.2	3.8
Congenital nephrotic syndrome	22	1.0	3.2
Mesangial proliferative glomerulonephritis	16	0.7	2.3
Membranous nephropathy—idiopathic	9	0.4	1.3
Alport Syndrome	8	0.4	1.2
Congenital nephrotic syndrome—FSGS	3	0.1	0.4
Minimal change	2	0.1	0.3
Nephrotic syndrome of childhood	2	0.1	0.3
Renal amyloid	2	0.1	0.3
Congenital nephrotic syndrome—innish type	2	0.1	0.3
IgM-associated nephropathy	1	0.0	0.1
Immunotactoid/fibrillary nephropathy	1	0.0	0.1

*n* number, *FSGS *focal segmental glomerulosclerosis, *RPGN* rapidly progressive glomerulonephritis, *MCGN* mesangiocapillary glomerulonephritis, *IgAN* immunoglobulin A nephropathy, *IgM* immunoglobulin M, *LN* lupus nephritis

**Table 3:** Frequencies and proportions of specific secondary glomerular diseases

Secondary glomerular disease	Frequency	Overall % (n = 2 135)	% of Secondary GN (n = 1 450)
Secondary	1450	67.9	100
DN	1198	56.1	82.6
HIVAN	102	4.8	7.0
LN	96	4.5	6.6
Glomerulonephritis—secondary to other systemic disease	15	0.7	1.0
ANCA-associated GN	11	0.5	0.8
Diffuse endocapillary glomerulonephritis	6	0.3	0.4
PAN	6	0.3	0.4
Anti-GBM disease	5	0.2	0.3
FSGS secondary to HIV	4	0.2	0.3
AA amyloid	3	0.1	0.2
Membranous nephropathy—infection-associated	2	0.1	0.1
FSGS secondary to obesity	1	0.0	0.1
Membranous nephropathy—drug-induced	1	0.0	0.1

*n* number, *FSGS* focal segmental glomerulosclerosis, *RPGN* rapidly progressive glomerulonephritis, *LN* lupus nephritis, *DN* diabetic nephropathy, *HIVAN* human immunodeficiency virus−associated nephropathy, *ANCA* antineutrophil cytoplasmic antibody, *GN* glomerulonephritis, *GBM* glomerular basement membrane, *HIV* human immunodeficiency virus, *PAN* polyarteritis nodosa

Ethnic distribution patterns varied within the cohort. Within our cohort of patients with glomerular disease, Black patients made up 49%, which is slightly less than the 52.5% of the total SARR cohort. Black patients only made up 37.2% of the patients with primary glomerular disease, much less than the 52.5% of the total SARR cohort. In contrast, when DN was excluded, Black patients made up two-thirds (66.7%) of the patients with secondary glomerular disease, notably more when compared to the overall SARR population. Patients of mixed ancestry, 17.2% of the total SARR population, made up 25.9% of those with glomerular disease, with primary glomerular disease accounting for 37.7% of cases in this group. Among Indian/Asian patients, 11.4% of the SARR population, who comprised 9.7% of the glomerular disease cohort, DN was particularly common, accounting for approximately 78.4% of glomerular disease cases (Tables 1, 4).

**Table 4:** A comparison of patients with common secondary glomerular diseases

	DN, *n* (%)	HIVAN, *n* (%)	LN, *n* (%)
Totals	1 198 (100)	102 (100)	96 (100)
Male sex	731 (61)	48 (47.1)	19 (19.8)
Age (years), median (IQR)	61 (54–69)	47 (41–53)	40 (32–52)
Ethnicity			
Black	624 (52.1)	101 (99)	36 (37.5)
Coloured/mixed	250 (20.9)	1 (1)	39 (40.6)
Indian/Asian	163 (13.6)	0	10 (10.4)
White	152 (12.7)	0	11 (11.5)
Province			
Western Cape	291 (24.3)	10 (9.8)	36 (37.5)
Gauteng	307 (25.6)	23 (22.5)	24 (25)
KwaZulu-Natal	230 (19.2)	13 (12.7)	8 (8.3)
Eastern Cape	181 (15.1)	9 (8.8)	10 (10.4)
Free State	44 (3.7)	29 (28.4)	8 (8.3)
Limpopo	62 (5.2)	3 (2.9)	3 (3.1)
Mpumalanga	30 (2.5)	13 (12.7)	2 (2.1)
North West	39 (3.3)	2 (2)	4 (4.2)
Northern Cape	14 (1.2)	0	1 (1)
Histologically confirmed	0	33 (32.4)	76 (79.2)
Healthcare sector			
Private	1 122 (93.7)	64 (62.7)	52 (54.2)
Public	76 (6.3)	38 (37.3)	44 (45.8)
KRT modality			
HD	1 062 (88.6)	80 (78.4)	64 (66.7)
PD	70 (5.8)	19 (18.6)	11 (11.5)
TX	66 (5.5)	3 (2.9)	21 (21.9)
Diabetes mellitus	1 198 (100)	5 (4.9)	9 (9.4)

*SARR* South African Renal Registry, *n* number, *IQR* interquartile range, *DN* diabetic nephropathy, *HIVAN* human immunodeficiency virus associated nephropathy, *LN* lupus nephritis, *HIV* human immunodeficiency virus, *KRT* kidney replacement therapy, *HD* haemodialysis, *PD* peritoneal dialysis, *TX* transplant, *DM* diabetes mellitus

Most patients with secondary glomerular disease (87.2%) received treatment in the private healthcare sector, whereas the majority of those with primary glomerular disease (60%) were treated in the public sector. This difference reflects the high prevalence of diabetic nephropathy within the secondary glomerular disease group, as most patients with DN (93.7%) were managed in the private sector (Table 4).

Overall, haemodialysis was the most common KRT modality in our patients (71.5%). Transplantation was more common in patients without diabetic nephropathy and most common among those with primary glomerular disease (41.2%); peritoneal dialysis was also more common among patients without diabetic nephropathy (16.3%) (Table 1).

The Western Cape accounted for the greatest proportion of the patients (62.8%) with primary glomerular disease, while Gauteng accounted for most of the secondary glomerular diseases (25.3%).

Table 4 provides a comparison of the three most common secondary glomerular diseases. The patients with diabetic nephropathy were much older (median age 61 years). Almost all of them (93.7%) were treated in the private sector. Most (88.6%) of patients with DN received HD as their KRT modality.

The group of patients with LN had a median age of 40 years. There was a significant female predominance (80.2%), and this diagnosis was most common in patients of mixed ancestry (40.6%). Most were residents in the Western Cape (37.5%) or Gauteng (25%).

The patients with HIVAN had a median age of 47 years, with a female predominance (52.9%) compared to the SARR cohort (41%). Regarding the ethnic distribution, most (99%) were Black. Very few of the patients in this group (2.9%) had a functioning kidney transplant.

Among people living with HIV (PLWH, Table S4), the majority were of Black ethnicity (94%) and most received treatment in the private sector (72.4%). There were almost equal proportions between the two sexes (50.4% male). The provincial distribution differed from the overall SARR cohort, with lower proportions from Gauteng and the Western Cape and higher contributions from the Free State, Eastern Cape, and Mpumalanga. There was a substantially lower rate of transplantation (4.7%) in PLWH compared to that of the overall SARR cohort (18.5%). Secondary glomerular diseases predominated (82.8%), with HIVAN being the most common diagnosis (53.1%), followed by DN (43.8%). Primary glomerular diseases were less frequent, accounting for 17.2% of cases. FSGS constituted 10% of the primary glomerular disease in the PLWH, higher than the 7.7% of primary glomerular diseases within the overall cohort (Table S5).

Three hundred and ten patients had a histologically proven diagnosis for their glomerular disease. This group, who were mostly female (54.7%), accounted for 24.5% of all the patients with primary glomerular disease and 9.8% of those with secondary glomerular disease. The majority were from the Western Cape (41.9%) and treated in the public healthcare sector (57.7%). Overall, LN was the most common diagnosis, while the most common primary glomerular disease was FSGS (Table S6).

## Discussion

This study provides a comprehensive overview of the burden and distribution of glomerular disease among patients receiving KRT in South Africa. Of the patients on KRT in 2022, 23.2% had glomerular disease as an underlying aetiology for their kidney failure (12.8% DN and 10.4% other glomerular disease), reflecting the significant contribution of glomerular disease to kidney failure in this population. This aligns with findings from previous studies in SSA, although regional patterns differ due to various demographic, socio-economic, and environmental factors [1, 3, 4].

Most of the South African population (85.4%) rely on the public healthcare sector for services, whilst only a small proportion (14.6%) have the means to seek care from the private healthcare sector [6]. More than half of our patients with primary glomerular disease were being treated in the public sector and, by contrast, most of those with secondary glomerular disease were being treated in the private sector for KRT, reflecting the burden of DN in this group of patients. Due to rationing of KRT in the public sector, resource limitations mean that patients with diabetes—especially those with multiple comorbidities such as established cardiovascular disease—may not be offered KRT, as cardiovascular disease remains the leading cause of mortality in this group. We have previously described how the selection process incorporates the accountability-for-reasonableness framework to support fairness and transparency [19]. In contrast, the private sector does not ration dialysis modalities. Nevertheless, not all patients with diabetes receiving dialysis privately proceed to transplantation, as transplant candidacy is determined by several considerations—including age and medical comorbidities—that may deem a patient high risk.

There are disparities in the provincial access to KRT, with the Western Cape having the highest treatment rates (290 pmp), followed by the Free State and Gauteng. This disparity, together with differences in access to kidney biopsy services, may lead to certain glomerular disease being overrepresented in certain provinces and underrepresented in others [6].

In South Africa, non-diabetic glomerular disease accounted for 10.4% of patients receiving KRT, a proportion comparable to that reported in several other regions [6]. Chronic glomerulonephritis contributes approximately 9.1% of kidney failure in the United States, 10% in Europe, and 12.7% in Sweden, with somewhat higher proportions observed in India (16%), Australia (18%), and New Zealand (19%) [20–24]. In contrast, substantially greater contributions are reported in Taiwan (25.1%) and China (61%) [25, 26].

DN, as a glomerular cause of kidney failure, appears less prevalent in South Africa, accounting for 12.8% of cases compared with 43.2% in Taiwan, 40–50% in Australia and New Zealand, 25% in India, 22% in Europe, 20% in Sweden, and 10–13.9% in the United States and China [5, 21–26]. By comparison, there is a low proportion of DN in the South African registry which is likely attributable to the selection criteria applied in the public sector [19, 27–29].

Consistent with prior registry findings, this study demonstrated a predominance of male patients (56.9%) and a median age of 55 years among those with glomerular disease receiving KRT. Similar age and sex distributions have been reported in other national registries, including ANZDATA, USRDS, and the UKRR, where glomerular diseases typically affect adults in early to middle adulthood and occur more frequently in males [6, 29–32]. This pattern indicates that glomerular disease in South Africa primarily affects individuals in their economically productive years, amplifying its impact on both patients and the broader health system.

Notably, secondary glomerular diseases accounted for two thirds of cases, a finding that deviates from studies in other regions where primary glomerular diseases are often more common [7]. This may reflect the high prevalence of systemic diseases such as diabetes mellitus and systemic lupus erythematosus in the South African population, along with the ongoing burden of infectious diseases such as HIV. An additional contributing factor is likely the limited access to kidney biopsies and genetic testing, particularly in the public sector, which may result in the underdiagnosis or misclassification of primary glomerular diseases. This is further supported by the high proportion of cases coded by clinicians as “GN without histology” (55.2% of all primary GN), suggesting that many patients may not have had a definitive diagnostic workup.

DN was the most common specific glomerular diagnosis, accounting for 56.1% of all cases, a pattern consistent with global trends [6]. Nearly all affected patients were receiving care in the private sector.

HIVAN accounted for 53% of secondary glomerular diseases among PLWH. Nearly all patients with HIVAN were of Black ethnicity (99%), with most from the provinces of Gauteng and the Free State, while KwaZulu-Natal, despite having the highest population HIV prevalence nationally, was relatively under-represented. This distribution likely reflects differences in access to KRT across provinces rather than underlying HIV prevalence alone. The median age of patients who had HIVAN was 47 years, and there was a slight female predominance, contrasting with the male predominance in the overall SARR cohort. This sex difference likely mirrors national HIV prevalence patterns, where HIV is much more common in females [33].

The concentration of HIVAN among Black patients may be partly explained by the high prevalence of APOL1 risk variants in individuals of African ancestry, which are strongly associated with susceptibility to HIVAN [9, 17]. In addition, the near absence of HIVAN in non-Black patients and the low number of FSGS cases attributed to HIV (3%) may reflect diagnostic misrepresentation due to limited biopsy rates, as well as evolving histopathological patterns in the ART era [17]. This shows that the epidemiology of HIVAN in patients receiving KRT in South Africa is shaped by both biological and health system factors.

LN was the third most common secondary glomerular disease in this cohort and was most frequently observed in patients of mixed ancestry and Black patients, with 37.5% originating from the Western Cape. It was also the most common diagnosis among patients with histologically proven glomerular disease. These findings are consistent with biopsy series from tertiary centres in South Africa, where LN has consistently emerged as the most prevalent secondary glomerular disease [8–11, 34–37]. Importantly, LN in South Africa is often characterised by aggressive histological patterns, with class IV LN predominating and associated with significantly poorer outcomes compared to international cohorts. In a large, three-decade-long review from the Western Cape, class IV disease accounted for more than half of cases and was linked to a five year survival rate of only 67%, markedly lower than outcomes reported elsewhere [38]. This reflects not only disease severity but also delays in presentation and limited access to KRT, which further worsen prognosis.

Among patients with primary glomerular disease, a large proportion (55.2%) were labelled as “GN without histology,” likely reflecting late clinical presentation and limited access to kidney biopsy services. Primary glomerular diseases were commonly recorded in younger adults and more frequently in the public sector. This may reflect differences in diagnostic practices and access to care rather than a true epidemiological distribution. Younger patients are more likely to undergo investigation for kidney disease, including biopsy, than older patients who often present late and with advanced CKD, leading to an over-representation in this group. The concentration of these cases in the Western Cape may also reflect regional differences in nephrology service availability and capacity in terms of both KRT and kidney biopsy practices. These factors may contribute to an overestimation of primary glomerular disease in this cohort.

A key limitation is that all diagnoses were supplied by the treating clinician or centre and were not independently confirmed or standardised across sites. Although the SARR encourages nephrologists to record “Cause unknown” when patients present late with small, shrunken kidneys, there is a long-standing practice of applying non-specific diagnostic labels such as “Chronic GN” or “Hypertensive kidney disease” in these scenarios. Such misclassification likely contributes to the observed distribution of primary glomerular disease and highlights the challenges of relying on registry-based clinical diagnoses, an issue not unique to South Africa. The overwhelming dominance of DN can further skew the interpretation of other glomerular diseases, particularly as there was no distinction in the registry as to how the diagnosis of DN was made.

From a histopathological perspective, LN was the most common secondary glomerular disease, whereas FSGS predominated among primary glomerular diseases. Biopsy series from the Gauteng and Free State provinces likewise report FSGS as the leading primary diagnosis, and together these provinces contributed nearly one quarter (22.6%) of all primary glomerular disease cases. Of the 53 patients with FSGS in this study, 32.1% and 9.4%, were from Gauteng and the Free State, respectively. The populations of both provinces are predominantly Black (84.6% and 88.9%, respectively) [14], a group more likely to carry high-risk APOL1 gene variants, which have been linked to the development of FSGS [39]. In contrast, MCGN cases were concentrated in the Western Cape, where people of mixed ancestry constitute the largest population group (42.1%) [14], therefore other environmental exposures (e.g., methamphetamine use) or underlying genetic susceptibility may predispose to this glomerular disease [4, 9, 11–13, 15, 16].

A key strength of this study is its national coverage and inclusion of both public and private sector data, providing a comprehensive overview of glomerular disease in patients receiving KRT in South Africa. However, limitations include potential diagnostic misclassification, underrepresentation of patients from rural provinces with limited access to kidney biopsy services and KRT, and the overwhelming predominance of DN, which may obscure trends in other glomerular diseases. The under-representation of rural provinces may reflect healthcare access disparities (KRT and kidney biopsy services) rather than true disease distribution. These factors highlight the need for improved diagnostic practices and access to histopathology to more accurately characterise the burden of primary glomerular diseases in South Africa.

## Conclusion

This study highlights the significant burden of glomerular diseases among South African patients receiving KRT, accounting for nearly one-quarter of all KRT cases. There are notable differences in distribution by age, sex, ethnicity, geography, and healthcare sector. Secondary glomerular diseases, particularly DN, HIVAN and LN were the predominant causes of kidney failure. Histologically proven cases suggest that LN and FSGS remain high-priority targets for further research, given their clinical impact and the potential for improved outcomes with earlier diagnosis and intervention. The data also highlight persistent inequities in access to KRT, with most patients with glomerular disease receiving care in the private sector. DN is especially notable for its strong association with the private healthcare sector, while primary glomerular diseases are more often managed in the public system.

There is a need for the establishment of a national kidney biopsy registry to complement the SARR and provide more detailed data on glomerular disease patterns.

## Supplementary Information

Below is the link to the electronic supplementary material.

Supplementary file1 (DOCX 22 KB)

## Data Availability

Data are available upon reasonable request and with the permission of the South African Renal Registry.
