# Supplementary material for: Glomerular diseases in South African patients on kidney replacement therapy: an analysis of 2022 South African Renal Registry data
Source: Int Urol Nephrol. 2026 Apr 2;58(10):4425–33. doi: 10.1007/s11255-026-05117-0 (PMC13586010; doi:10.1007/s11255-026-05117-0)
Supplement: Supplementary file 1 [file 11255_2026_5117_MOESM1_ESM.docx]

**Supplementary tables**

**Table S4.** **A comparison of people living with human immunodeficiency virus (PLWH).**

|  | **SARR, (%)** | **HIV Patients, n (%)** |
| --- | --- | --- |
| **Total, n (%)** |  | 232 |
| **Sex** | | |
| Male | 59 | 117 (50.4) |
| **Age (years), median (IQR)** | 54 (43-64) | 49 (42-56) |
| **Ethnicity** | | |
| Black | 52.5 | 218 (94) |
| Coloured/Mixed | 17.2 | 7 (3) |
| Indian/Asian | 11.4 | 4 (1.7) |
| White | 17.1 | 2 (0.9) |
| Other |  | 1 (0.4) |
| **Province** | | |
| Western Cape | 23.1 | 34 (14.7) |
| Gauteng | 30.2 | 54 (23.3) |
| KwaZulu-Natal | 17.7 | 37 (15.9) |
| Eastern Cape | 11 | 38 (16.4) |
| Free State | 6.6 | 43 (18.5) |
| Limpopo | 3.8 | 5 (2.2) |
| Mpumalanga | 2.6 | 18 (7.8) |
| North West | 3.2 | 3 (1.3) |
| **Histology** | | |
| Yes |  | 48 (20.7) |
| **Sector** | | |
| Private | 74.6 | 168 (72.4) |
| Public | 25.4 | 64 (27.6) |
| **KRT modality** | | |
| HD | 71.7 | 182 (78.4) |
| PD | 9.8 | 39 (16.8) |
| TX | 18.5 | 11 (4.7) |
| **DM** | | |
| Yes | 31.9 | 91 (39.2) |

**^Abbreviations:^** ^SARR (South African renal registry), HIV (human immunodeficiency virus), KRT (kidney replacement therapy), HD (haemodialysis), PD (peritoneal dialysis), TX (transplant), DM (diabetes mellitus).^

**Table S5. Frequencies and proportions of specific primary and secondary glomerular diseases in people living with human immunodeficiency virus (PLWH).**

| **Glomerular disease** | **Overall % Primary GN**  **(n=685)** | **% of Primary GN**  **in PLWH**  **(n=40)** |
| --- | --- | --- |
| **Primary** | 32.1 | 17.2 |
| Glomerulonephritis - no histology | 55.2 | 50 |
| FSGS | 7.7 | 10 |
| IgAN | 5.3 | 7.5 |
| RPGN | 4.8 | 5 |
| Membranous nephropathy - idiopathic | 1.3 | 5 |
| Glomerulonephritis - histologically indeterminate | 7.2 | 2.5 |
| Adult nephrotic syndrome - no histology | 6.1 | 2.5 |
| Mesangial proliferative glomerulonephritis | 2.3 | 2.5 |
| Nephrotic syndrome of childhood | 0.3 | 2.5 |
|  | **Overall %**  **Secondary GN**  **(n=1450)** | **% of Secondary GN**  **in PLWH**  **(n=192)** |
| **Secondary** | 67.9 | 82.8 |
| HIVAN | 7 | 53.1 |
| DN | 82.6 | 43.8 |
| FSGS secondary to HIV | 0.3 | 2.1 |
| Glomerulonephritis - secondary to other systemic disease | 1.0 | 2.1 |
| LN | 6.6 | 1.0 |
| ANCA-associated GN | 0.8 | 0.5 |

**^Abbreviations:^** ^FSGS (focal segmental glomerulosclerosis), RPGN (rapidly progressive glomerulonephritis), IgAN (immunoglobulin A nephropathy), LN (lupus nephritis), DN (diabetic nephropathy), HIVAN (human immunodeficiency virus-associated nephropathy), ANCA (antineutrophil cytoplasmic antibody), GN (glomerulonephritis), HIV (human immunodeficiency virus).^

**Table S6. Frequency of histologically proven primary and secondary glomerular diseases.**

| **Primary** | **Frequency** | **%Primary**  **(n=168)** |
| --- | --- | --- |
| FSGS | 52 | 31.0 |
| RPGN | 33 | 19.6 |
| MCGN | 26 | 15.5 |
| IgAN | 19 | 11.3 |
| Mesangial proliferative glomerulonephritis | 16 | 9.5 |
| Membranous nephropathy - idiopathic | 9 | 5.4 |
| Alport Syndrome | 3 | 1.8 |
| Congenital nephrotic syndrome- FSGS | 3 | 1.8 |
| Congenital nephrotic syndrome- Finnish type | 2 | 1.2 |
| Renal amyloid | 2 | 1.2 |
| IgM - associated nephropathy | 1 | 0.6 |
| Immunotactoid / fibrillary nephropathy | 1 | 0.6 |
| Minimal change | 1 | 0.6 |
| **Secondary** | **Frequency** | **%Secondary**  **(n=142)** |
| LN | 76 | 53.5 |
| HIVAN | 33 | 23.2 |
| ANCA-associated GN | 7 | 4.9 |
| Diffuse endocapillary glomerulonephritis | 6 | 4.2 |
| PAN | 6 | 4.2 |
| FSGS secondary to HIV | 4 | 2.8 |
| Anti-GBM | 3 | 2.1 |
| AA amyloid | 3 | 2.1 |
| Membranous nephropathy - infection associated | 2 | 1.4 |
| FSGS secondary to obesity | 1 | 0.7 |
| Membranous nephropathy - drug-induced | 1 | 0.7 |

**^Abbreviations:^** ^FSGS (focal segmental glomerulosclerosis), RPGN (rapidly progressive glomerulonephritis), MCGN (mesangiocapillary glomerulonephritis), IgAN (immunoglobulin A nephropathy), IgM (immunoglobulin M), LN (lupus nephritis), HIVAN (human immunodeficiency virus associated nephropathy), ANCA (antineutrophil cytoplasmic antibody), GN (glomerulonephritis), PAN (polyarteritis nodosa), HIV (human immunodeficiency virus), GBM (glomerular basement membrane).^
